# Eclampsia Parturientum*Read before the Boston Gyneological Society.

**Published:** 1884-04

**Authors:** Frank S. Billings


### THE JOURNAL

OF

### COMPARATIVE MEDICINE AND SURGERY.

VOL. V. APRIL, 1884. No. 2.

### ORIGINAL COMMUNICATIONS.

### ART VIII.—ECLAMPSIA PARTURIENTUM.*

BY FRANK S. BILLINGS, V. S.

IF there is a single disease, or condition in the whole medical nosology, which with regard to its etiology may be said to be of a purely theoretic nature Eclampsia is that one. If there is any one subject to which man can bend his whole energies and still feel he knows as little as when he began to study it, Eclampsia is that one.

Derivatively speaking, the word Eclampsia has but very little connection with the condition we are about to discuss. According to Dunglison it means "flashes of light before the eyes;" or vulgarly speaking "seeing stars." In reality the word expresses but a single phenomenon of many, yet usage has made it apply to convulsions, more particularly those of a certain nature which occasionally occur during pregnancy or parturition This conception is also erroneous as we know that exactly similar phenomena occur under circumstances that have no connection either with gravidity or parturition, as in Scarlatina, Diptheria, Cholera, etc. We shall see that this fact is of very great etiological importance.

* Read before the Boston Gyneological Society.

It would be well if we could drop the word Eclampsia and in its stead speak of Convulsions in parturition.

*Eclampsia is not a disease, neither is it a diseased condition of an organism.*

*Eclampsia represents a result; the outward visible phenomena by which certain internal disturbances manifest themselves to the observer.*

DEFINITION.

There appears to be far more uniformity of opinion among medical men, than Veterinarians as to the nature of this complication. If one desires to read it up in Medical works, he can always find it under the name "Eclampsia," while in Veterinary medicine he would be troubled to know what he was seeking for, or where to find it, so many names has it enjoyed. The most common of these are milk fever; calving fever; parturient fever, parturient apoplexia, parturient collapse, etc. Franck is the only author that looks upon it as identical with Eclampsia in woman and is in fact, the only Veterinary author that has given much study to the subject.

Franck defines Eclampsia: "as a very acute and dangerous disease, which, in general, appears soon after parturition, especially in cows, and in them chiefly such as belong to the great milk yielding breeds; the disease is characterized by an extreme degree of unconsciousness, and paralysis, seldom by convulsions, *and is due to anaemic conditions of the brain.*

Mr. Fleming does not accept Franck's nomenclature, but calls the condition "Parturient collapse," and not Eclampsia, "*because in women it is essentially epileptic* (more correctly epileptoid), *or convulsive, though in the cow convulsions sometimes occur, yet they are far from being a constant symptom.*"

We cannot see how Fleming can logically disagree with Franck, for his treatment of this subject is a literal translation of the latter; he thereby adopts Franck's theory as to the cause of the condition, which the latter adapted from human medicine, and hence logically assumed the same name for complications which he looked upon as etiologically alike, though occurring in different species under similar circumstances. Fleming contradicts himself, for in another place we may read, that though the disease in cattle is not so characterized by convulsions still "we do not dispute that the same, or similar, causes may operate in the production of both;" again, "the anatomical arrangement of the 'rete mirable' is such that it chiefly supplies the cerebrum; *this should tend to explain why in Eclampsia in the Cow, the comatose symptoms are so common that convulsions seem exceptional.*" If the condition is not 'Eclampsia,' why speak of it as such all through the text, and why deny it in one place and shortly afterward accept it again?

The reason that convulsions are not so frequent or severe in cows, assuming the cause to be the same, is surely not to be sought as much in any peculiarities of the circulation, as in the very low degree of nervous irritability which is common to the bovine species.

Williams speaks of Eclampsia, "as Parturient apoplexia" and defines it "as a disease characterized by suppression of the lacteal secretion and apoplexia."

Roell looks upon it as "Calving fever," and "a disease which is characterized by disturbances in the digestive track and sudden paralysis."

ECLAMPSIA IN WOMAN.

Spiegelberg (Geburtshülfe) says: "*with the name 'Eclampsia' authors have designated epileptoid convulsions of a tonic but more especially clonic character, accompanied by unconsciousness and coma, which occur in the course of the puerperium and are intimately connected with it.*"

I would call especial notice to the language of this definition, it is a model, it simply tells us what Eclampsia is considered to be, but does not mention anything with reference to cause, *nor is it defined as a disease.*

Notnagel (Ziemssen Nervenkrenkheiten) considers Eclampsia to be acute epilepsia, and that the majority of cases defined as E. parturientum should not be looked upon as such, although isolated cases of convulsions during pregnancy and parturition must be considered as truly Eclamptic. These are cases, in which we can find no indications of Albuminuria, or such in which the latter develops subsequent to the appearance of the convulsions.

Wernick endeavors to connect Eclampsia with injury to the Ischiatic nerve caused by the enlarged uterus: and considers these ischiatic lesions to act in the same way as injury to the nerve does in rabbits; that is, cause excitability of the

Pons and Medulla oblongata. If to this be added the irritation of other peripheral nerve regions, such as the sensitive nerves of the sexual organs (intra partum), then Eclamptic convulsions follow.

### Course and Phenomena in Woman.

Eclampsia * may occur in all phases of the puerperium, frequently during the act of birth, but generally the eruption follows quickly on the birth of the child, occasionally several days may elapse before it takes place. It seldom occurs before the seventh month of pregnancy, though quite a number of cases are recorded during the sixth month, and occasionally one in the fourth or fifth.

In the majority of cases prodromal phenomena anticipate the convulsions; these generally consist of gastric and sensorial disturbances, œdema of the face and extremities, such as headache, vertigo, depression, *Amaurosis*, dyspepsia, emesis, etc. Oedema of the hands and face is essentially suspicious.

Eclampsia is manifest in the character of the first attack, which is that of an epiliptic convulsion, except that it is of a more intense nature. Tonic and clonic convulsions of a most variable nature follow each other indiscriminately, so that we cannot logically speak of a protopathic, tonic, and a deuteropathic, clonic, stage. The patient is utterly unconscious.

The outlines of the face become changed. The eyelids are rapidly closed and opened; the bulbus moved in every direction, or fixedly directed upwards or to one side; the pupil is distended and not easily excited. The mouth is frequently distorted; *the teeth gnashed and ground together*, often catching the tongue between them and injuring it. The head is drawn to one side, tetanically, *or bent in the neck.* The convulsions extend to the whole body; Opisthotonos is frequent; the respiratory muscles partake in the convulsions when at their height causing momentary cessation of the respiration. The face, at first pale, soon becomes livid, and cyanotic; the conjunctivae are injected; the carotids beat heavily; the pulse is at first small, weak, frequent, often, intermittent. The temperature is elevated. The storm relaxes in severity in a short time, the convulsions ceasing suddenly, or the rest being at

* Spiegelberg—Ibid.

first interrupted by a few short ones. The eye-lids close; bloody foam issues from the mouth, the lips and tongue being bitten and swollen, the respiration becomes easier. Cheyne-Stokes phenomena being frequently presented: the pulse becomes stronger and slower; the temperature rises still more; the face is cyanotic and assumes an idiotic expression; the patient lies in a soporous condition, being disturbed occasionally by *contractions of the uterus.* The sopor is not very profound at first; if the period of rest is of long duration consciousness, may return, but this is less likely to occur the more stormy the convulsions, and does not return if the latter have been several times repeated.

The duration of the convulsions seldom extends over 10-30 seconds: a much longer duration is incompatible with the continuation of life on account of the coeval complication of the respiratory muscles.

The period elapsing between the convulsions is of variable length; it is seldom that we see the trouble terminate in a single, or in very few convulsions. The more intensive their character the more frequently do they recur, and their number may be exceedingly great; authors have reported counting as many as 145-160 consecutive convulsions.

Exceptional phenomena during the outbreak of puerperal Eclampsia are periods of excitement during the intervals of quiet, the patient being totally unconscious at the same time; paralysis and twitching of the muscles of one side; awaking from the coma after a succession of convulsions, to sink into it still more profoundly with the next eruption.

*Authors have frequently assumed a causal connection between the pains and the convulsions*, Kuivisch having asserted that "Eclampsia never occurs without pains." This is erroneous as is proven by many observations of Eclampsia during pregnancy or by a completely quiet uterus: nevertheless it is a fact that eruptions of Eclampsia during gravidity are soon followed by parturition.

The deportment of the Urinary secretion demands especial consideration. In nearly every case it is strongly albuminous, and contains renal epithelium, (frequently filled with fatty detritus), sometimes cylinders, mostly fibrinous, seldom blood: the urates are often found to be decidedly diminished in quantity. In all severe cases the secretion of urine is very small, in fact the bladder may be entirely empty for several hours—anuria.

### Course and Phenomena in Cattle.

The outbreak of the disease is very sudden, most frequently within 12 to 15 hours from the time of parturition; occasionally two or three days may elapse before it occurs. Some have asserted that the disease generally appears with the recurrence of the pains by which the secundines-placenta are expelled. Eclampsia can occur before and during the parturition or under conditions which have no connection with it. It can also occur in other animals than cows, as oxen, dogs, swine, entirely independent of, or in females, as in the cow, in case of parturition.

It is universally admitted, and herdsmen will always tell you, that the birth was very easy in such cases; in fact Williams says, that he never knew of it occurring in a cow when the parturition was protracted and labored.

The prodromal phenomena generally escape attention; the first that come to observation are loss of appetite, a peculiar form of uneasiness and uncertainty in movement: striking at the abdomen with the hind legs as if in pain; tenesmus and shivering. Sometimes, phenomena indicative of hyperaemia cerebri are present early in the disease; the patient presses its head against the wall of the stable, or stall, bellows, grinds its teeth and is very violent, the eyes are fixed, at first watery, finally dry, retracted, expressionless, amaurotic. These early phenomena are of short duration.

With the progression of the internal conditions, the phenomena increase in intensity; the animal cannot control its movements, and after repeated trials either lies or falls down; convulsions sometimes occur. A soporous condition soon develops which is that in which the practitioner generally finds the animal at his visit. The position which the patient now assumes can be said to be pathognomonic. It lies prostrate with one hind leg doubled under the body, the other extended, sometimes both; the head is sometimes extended, but more frequently thrown back on the shoulder; a position it immediately falls into again, like a lump of lead, if we endeavor to move it. The patient is utterly unconscious of its surroundings and seems to groan irrespective of any pain. The pulse is at first excited and easily to be felt, but soon becomes indistinct and very rapid, 120 beats per minute or more; this acceleration of the pulse would seem to indicate paralysis of the vasa-motor centrum of the Vagus. The respiratory phenomena show little change at first, but gradually increase to 80 or 90 per minute; but with the extension of the disease the respiratory movements become slower and labored.

The digestive tract becomes rapidly complicated. The oral mucosa is pale; paralysis of the canal from the pharynx to anus follows, with complete retarding of defecation; the fœces in the rectum become dessicated and hard. The secretion of saliva continues and on account of inability to swallow accumulates in and flows from the mouth. It is dangerous to give medicine by the mouth, foreign-body pneumonia frequently following. Tympanitis is frequently present, causing an eructation of gases and elements of food, which getting into the pharynx are also a cause of foreign-body pnuemonia. The secretion of urine generally diminishes early in the disease; anuria is frequently present. The urine contains albumen and casts. Franck says he has frequently observed albumen to be present in the urine of cows antecedent to parturition.

Veterinarians do not seem to have given sufficient attention to this condition of the urine, a circumstance which must necessarily lead to mistaken views as to some of the complications, in fact that which we consider the chief one of the disease.

That albuminuria is present in nearly every severe case is unquestionably true, yet so practical a writer as Williams says: "*the urine is pale in color and free from albumin; the secretion is retarded.*"

Fleming says: "Micturition is also, as a rule, suspended from the commencement, consequently urine accumulates in the bladder:" when speaking of treatment he also says: "the urine should be frequently removed from the bladder."

Here are two errors, one due to false translation; the other to want of practical experience, or perhaps forgetfulness.

Micturition means passing of urine. Mr. Fleming should have translated Franck's words as follows: "the secretion of urine is generally stopped, Anuria; in some cases, it is only partially suppressed, however."

Urine does not accumulate in the bladder, that organ being frequently almost empty for hours, hence removal of its contents is unnecessary unless at an early stage, or in order to examine it. On account of the paralyzed condition of the patient it is a question if the urine would not flow out ot itself, the position of the animal favoring it, when the bladder over fills, Spinola says, "that it frequently does." I have never seen a bladder so distended, even in the early stages, that it was necessary to empty it for the comfort or health of the animal. The secretion of milk is also checked, frequently entirely.

The general irritability of the nervous system is entirely wanting. The lachrymal secretion is at first profuse, but finally ceases entirely; a return of it is said to be a favorable indication. Amaurosis exists, but no opthalmoscopic study of the eyes has ever been attempted.

The temperature of the body is always lower than normal, hence the term fever is obviously improper. No one seems to have noticed whether it rises in cattle when convulsions are present. In fatal cases these conditions simply go on augumenting in intensity.

### RESUMÉ.

| MAN. | CATTLE. |
|---|---|
| The condition is essentially one of parturition, but may occur in the course of other diseases. | While Eclampsia seems to be almost, if not entirely limited to parturition in cows, it is very seldom that it occurs in other animals at that time or any other. According to my experience the dog is more subject to it, independent of parturitions than any other animal. |
| Prodromal phenomena are generally reported. | On account of the impossibility of subjective examination of patients we cannot say anything about such phenomena, but it is very certain that some such sensations as common to man, must be present in animals. |
| Amaurosis is almost always present | Amaurosis is always present in cows. |

| | |
|---|---|
| Oedema of face and extremities. | Not present. |
| Characterized by Convulsions. | Convulsions seldom in cattle, very frequently seen in dogs. |
| Patients grind their teeth. | Cows do the same. |
| Head frequently drawn to one side by contraction of muscles of neck. | Very characteristic in Cows. |

Pulse similar in each as well as Respirations.

| | |
|---|---|
| A rise of temperature occurs. | Temperature is below normal. |
| Secretion of Urine suddenly ceases. | Same in cattle. |
| Albumin and Casts generally present. | |
| Bladder contains little Urine. | Same in cattle. |
| Cannot find anything with reference to it. | Milk secretion suddenly diminishes and frequently ceases almost entirely. |
| Same as above. | Complete paralysis of digestive tract, cessation of defecation, dessication and stocking of fæces in rectum |
| Same as above. | Tympanitis. |
| do do | Pneumonia frequently follows in consequence of eructated particles of food getting into the trachea. |

### Etiology.

It is not necessary for us to consider the many theories which have been advanced by different authors as to the cause of Eclamptic convulsions for a careful study of this question reveals the fact that all observers of note are advocates of one or two theories. It is not my intention to go into all the evidence pro and con for either of these, but to state them as succintly as possible in order to lead others to consider them and especially to call the attention of observers to the fact that *one cause must produce the phenomena in man and animals.*

These two theories are :

1st. That Eclampsia is due to Oedema cerebri and the sequential Anaemia.

2d. That it is due to intoxication of the blood with the entire element of the organism which go to make up the urine.

At the present day nearly all the most competent observers in human medicine favor the latter theory.

Aside from these supposed direct causes there are various conditions of the organism, in cattle especially, which play a very essential role in the generation of Eclampsia, of these we shall treat presently.

As the name indicates parturition plays a most important part in the generation of eclampsia. Irrespective of the direct theoretic cause I think it can be safely asserted that eclampsia has never been seen in cows except in connection with parturition, though cases have been reported in swine, goats, and oxen (?); but singular to say little mention is made of dogs in which eclamptoid (at least) convulsions due to urine-intoxication is no uncommon occurrence. The theory that aedema cerebri and the sequential Anaemia is the cause of Eclampsia is otherwise known as the Traube-Rosenberg theory.

For Veterinary Medicine it is sufficient to say that Franck, the best authority on Animal Obstetrics, has adopted and very ably defended it. Mr. Fleming's work on the same subject is but a literal translation of Franck's.

The latter says: "The disease is due to Anaemia of the brain." He postulates the T. and B. theory as follows:

*a.* The cause of the eclamptic phenomena are to be sought in changes of the circulation in the brain, and not in uraemic blood intoxication.

*b.* These changes in the cerebral circulation are due to augumented blood pressure in aortic circulation system, which is largely caused by the sudden, and violent contraction of the uterus in the cow (which is peculiar to these disturbances) in consequences of which a large organic system is thrown out of circulation, and a hyperaemic condition caused in other organs; this hyperaemia in the brain, by the pressure it exerts on the one side and the contrary force exerted by the calvaria on the other soon induces Oedema cerebri which is followed by Anaemia.

*c.* The development of Oedema and Anaemia of the brain under the circumstances in question finds essential support in the hydraemic condition of the blood which is present in gravidity. *This hydraemia is frequently increased by the Albuminuria which often occurs during the latter days of pregnancy.*

The full meaning of the last assertion appears to have entirely escaped Franck's attention, for he does not seem to have sought for any cause for the Albuminuria, or to have reflected upon the fact that Anuria frequently exists. Had he done so we do not think he could have so positively denied any causal nexus between Uraemic-intoxication and eclampsia.

The T. B. theory can also be very briefly stated as follows: To Anaemia cerebri three conditions are necessary:

1. Hydraemia.

2. Augmented Aortic pressure.

3. Accidental causes which in one way or another tend to support the action of the first two conditions.

OBJECTIONS.

1st. Phenomena completely identical with those appearing in connection with the puerperium and entirely independent of Oedematous conditions in the brain occur in other diseases, such as Cholera, Scarlatina, and Diptheritis; in all these cases Nephritis, or in other words, Eclampsia is due to the influence of the intoxication of the blood with the elements of which the urine is composed in toto. That Eclampsia can occur entirely independent of hydraemia, or increased aortic pressure, the two principal causes of Oedema and Anaemia cerebri is directly proven in cholera, in which the opposite conditions occur. While in cholera, scarlatina, etc., the Eclamptic phenomena are due to the results of nephritis—hence urine intoxication—it is equally well known that similar convulsions can also occur in nephritis, though every case of the latter is not necessarily complicated in this manner. To the development of the convulsions an unknown something is necessary or else they would be a universal accompaniment of urine-intoxication. This may be either prolonged action upon the nervous elements, or acute and intense action.

We have almost imperceptibly drifted into the consideration of the second theory; which is known as the Uraemic, or more correctly speaking Urine intoxication theory.

It must be born in mind that it is not the urates alone, but the fluid elements as well as all other salts which are excreted by the kidneys and form the urine that are looked upon by the advocates of this theory, as the chief cause of Eclampsia.

A point which seems to have escaped the attention of observers and which I am sure is not without some value, especially in very severe cases and in the later stages is the effect of the semi-paralized condition of muscles of respiration which must certainly seriously interfere with the proper oxidation of the blood.

As Mr. Fleming's work is the only one of importance accessible to my veterinary colleagues who read only English, and as the theory of urine intoxication is hardly touched upon there, and further as it has the best medical authors as its advocates, I may be excused for offering translations, in part instead of expressing the ideas of others in my own words.

By far the best treatise I have read upon the subject is in Spiegelburg who says: "Among the diseased conditions which have aetiological connection with puerperal eclampsia, only one is known that has the property connected with it of producing the peculiar convulsions by which this disease is characterized, and that is *existing or produced disease of the kidneys* by which urine intoxication " Harn intoxication, or Uraemia is produced." "I look upon Eclampsia as essentially a puerperal condition dependant upon urine intoxication." "The urine deserves especial consideration. It is, in general *very albuminous*, and contains renal epithelium and cylinders, the cell often displays fatty degeneration of their plasma: blood is sometimes found mixed with the urine: I have several times observed that the urates were strikingly absent in the urine. In all severe cases, the quantity of urine is found to be much diminished; the bladder has been found to be entirely empty for several hours at a time, Anuria."

"The etiology of Eclampsia only obtained a firm foundation with the proof that it was almost always connected with marked disturbance of the renal functions. (Lever 1843) was the first to demonstrate the albuminous condition of the urine in Eclampsia, and that in the kidneys of persons perishing from the disease, changes similar to those found in Bright's were frequently to be seen, hence it was concluded that the convulsions of Eclampsia as those of Uraemia in Nephritis must be caused by the retention of the urates in the blood." With reference to Friererick's theory that the convulsions are not directly due to the action of the urates in the blood, but that they became changed into carbonate of Ammonia, Spiegelberg says "Ammonaemia is to be looked upon as one of the most sure causes of Eclampsia." "At present we know that the convulsions are not caused by the action of any single element of the urine, *but by the retention in the blood of all the effete materials which find their excretion by means of the kidneys.*" "It is self-evident that the sudden interruption of the secreting functions of the kidneys, and the consequent retention of all these effete elements in the blood and tissues must exert a most injurious effect upon the diseased organism and cause the convulsions in Eclampsia. The proof that such elements must be present and retained in the blood and tissues is to be sought in the diminished quantity of urine secreted, which may even extend to Anuria, and the marked increase of the urates, as I have myself found in several cases on personal examination of the blood. Such an acute insufficiency of the kidneys may occur in persons that have previously enjoyed good health. This fact, and also that in some cases of Eclampsia no sign of disease in the kidneys could be discovered has led authors to doubt the correctness of the urine-intoxication theory. The intoxication of the blood with urates, etc., does not necessarily depend upon the actual existence of disease in the kidneys. Sudden cessation of the secretion of urine by the kidneys is dependent upon disturbances of the circulation in those organs; *i. e.*, upon a quickly developed complication of the renal blood vessels; the same need not necessarily have any evidence capable of discovery at an autopsy, and can disappear as rapidly on recovery as they appeared in the disease. In this way only can we understand those cases in which all renal symptoms vanish with the disappearance of the convulsions, and complete recovery follows with no indications of renal disease."

"We can easily explain why the convulsions are caused by disturbances of the renal blood vessels in all these cases were diffuse disease of the kidneys has not previously existed. Venous congestion, caused by pressure upon the renal veins by the pregnant uterus is scarcely probable, as we seldom find any intimation of congestion in the kidneys of persons that have died in Eclampsia; in fact these organs are most frequently found in an anaemic condition. *The cause must be sought in the*

*blood vessels, and especially at the terminations of the arterioles.* It can consist in a contraction of the vascular walls which suddenly shuts off the supply of blood to the secreting elements of the kidneys, hence in this case the primary cause must be sought in an irritation of the vasa motor nerves. The sensitiveness of the secreting epithelium of the kidneys, even to a short interruption of the blood supply, is well known ; the secretion of urine ceasing for a long time (3 quarters of an hour) if the circulation in a renal artery is only stopped for a moment. A regular and undisturbed supply of blood is indispensible to the secreting activity of the renal epithelium. A sudden and protracted contraction of the arteries of the kidneys would therefore be followed by cessation of their secreting functions and the accumulation of the element of the urine in the blood. Genuine Eclampsia is then caused by urine intoxication, in consequence of insufficiency of the secreting functions of the kidneys." Those cases of Eclampsia which occur independent of albuminuria belong to a special group, being acute epileptic convulsions. The epilepto-genetic zone, is to be sought within the limits of the ischiaticus.

Cohnheim (Pathologie vol. 2 p. 306) says, with reference to the connection between a contracted condition of the arterial walls in the kidneys and Eclampsia, "it is further of great importance to know that the diminution of the urine secreted before and during the convulsions in Eclampsia Parturientum is dependent upon a contracted condition of the arterial walls. Any one that has had frequent opportunities to examine the kidneys of women that have perished during the convulsions of Eclampsia knows that, at least in the acute cases, not a sign of nephritis is to be seen. The assumption that a highly congested condition of the kidneys is present, due to pressure of the pregnant uterus, finds its contradiction not only on topographical grounds, but also in the fact that the kidneys in Eclamptic cases are, as a rule, pale, soft, and not aedematous which should be the case were hindrances present to, the veinous outflow of the blood from these organs, it is not so in this disease. "The Malpighian bodies in the kidneys are very sensitive to disturbances of the circulation. Herman was the first to observe, and Overbeck confirmed it, that a short interruption of the arterial circulation in the kidneys was followed by complete cessation of urine secretion, and that when the kidneys again began to secrete urine the latter contained blood cells and albumin. In the same way the urine contains albumin when the secretion again begins after attacks of cholera : it also occurs in women on recovery from Eclampsia." As my paper is drawing to an alarming and unexpected length I will simply state that Huguenin, Bartels and Thomas (Ziemssen) are advocates of the urine intoxication theory as the chief cause of Eclampsia, as is also the case with Israel-Berlin-Klin, Wochenschift, Lohlein, Senator, Felz and Ritter, Rheinstaedler and others. (Ibid. 1878, 79, 80, 81) Klebs, Birsch, Hirschfeld and Samuels also stand upon the same ground.

Notnagel takes a somewhat different ground, proposing to limit the designation Eclampsia to those cases of Epileptoid spasms which occur independent of any certain organic disease, as acute idiopathic acute disturbances due to reflex excitations.

An English Veterinarian, Cox, has lately come out with a theory which has neither sense, facts, or pathological anatomy to stand upon, it is that "Eclampsia (in cows) is due to thrombi being formed in the cotyledon of the uterus, and that the phenomena of the disease are due to Embolism and its effects." To which may be answered that a sudden and powerful contraction of the uterus, common to this disease, is not consistent with thombosis and embolism, and that every indication of acute embolic infarction is wanting at necroscopical examinations.

### PREDISPOSING AND SUPPORTING CIRCUMSTANCES CONNECTED WITH PARTURIENT ECLAMPSIA IN COWS.

In this connection we have yet to consider quite a number of conditions in the cow which practical observation has demonstrated play a not unimportant role in connection with Eclampsia that I desire to call to the attention of my medical colleagues in the interest of comparative medicine. In this connection I must say that the conditions of the patient and the symptoms during the existence of the disturbance are much better described byFranck, with reference to the cow, than is the case with medical writers with regard to woman.

I quite agree with Spiegelberg, Cohnheim and others that antecedent Nephritis is not a necessary etiological moment, for while it may play a part in woman, it is seldom present in the cow.

Eclampsia is always connected with a quick and easy parturition in the cow, authors all agreeing that protracted and labored acts are never followed by convulsions.

It is also always connected with a speedy and powerful contraction of the uterus.

It seldom occurs before the secundines have been delivered.

It is an evil which seems to belong especially to the noted milk producing breeds.

In this regard something more is necessary than that a cow be even a milk machine for the old fashioned coarse-hipped cow of New England is a big milker, or can be made so by feeding, but she keeps thin "runs to milk," and we scarcely ever see a case in such cows. Eclampsia is essentially a product of refinement in breeding. It is as certainly connected with overfeeding, and an overfat condition of cows of the fine bred, milk and butter producing breeds. It is also a question if the nervous organization has not also a higher degree of development in these cows than in the beef producing breeds and the so called common cattle.

I have seen Eclampsia follow parturition in pure bred or grade cows more frequently when they calved in early summer, and had been injudiciously turned out to graze all they could on the fat and juicy grasses of May and June, than in the winter or spring months when stall-feeding was all they had. That Eclampsia is intimately connected with the condition of milk-production seems indicated by the fact that more cases occur in cows at an age when their milk producing functions are at the height of their development than at any other period. It seldom occurs with the first or second parturition, and diminishes in frequency after the seventh or eighth.

### Conclusion.

After carefully reviewing the two prominent theories with regard to the etiology of Eclampsia, in so far as they have been considered conformable with the symptoms of the disease, still we cannot but admit that neither of them completely satisfies our mind. We can but feel that something is still wanting to complete the history of this severe disturbance.

The fact that diffuse nephrites can and frequently does occur without any appearance of convulsions, that Anaemia cerebri may also be due to other causes than hyperaemia and oedema of the brain renders it evident that in parturient eclampsia some other factor must play an essential part in such cases, and that there must be in such patients some peculiar condition, or idiosyncrasy, present at such times, that is not present, even in the same patients at others, for our literature is full of reports of cases of Eclampsia which occurred at one parturition, but never recurred at succeeding ones. The question then is, what is this condition, or factor. To this we must answer that it must be some peculiar condition of the nervous system, perhaps some irritable connection between the uteral and renal plexus, which by reflex action extends to the brain, or other nervous centres.

If this is so, and personally, I can see no reason to doubt it, then eclampsia is essentially a

### Parturient Neurosis.

We have seen that Spiegelberg and Cohnheim both favor the view that convulsive contraction of the walls of the arteries in the kidneys can, and does produce a retention of the elements of the urine in the blood. What produces this condition in the kidneys? The parturient act, or when Eclampsia occurs in gravidity, a similar irritability of the uteral plexus.

If this is so, is there any just reason to doubt that by reflex action, a similar convulsive contraction of the cerebral arteries can also occur, which would naturally result in Anaemia cerebri, though not in the sense of the theory advocated by Franck.

The same reflex action may also extend to the blood vessels of the lungs and thus interfere with the non-oxidation of the blood.

When present, urine-intoxication undoubtedly plays a most serious part in the etiology of Eclampsia. The question still is, is it always present, notwithstanding the fact that albuminuria is often wanting. The answer to this question would seem to be in the affirmative, for diminution, or cessation, of the secretion of urine is a constant phenomenon, the result of which must be that the elements of the urine must be retained somewhere, and this somewhere must be in the blood and tissues, so that we are forced to the conclusion that Eclampsia parturientum is due to two causes:

1st. A peculiar irritability of the uteral plexus, producing a neurosis, which acts:

2d. Upon the arterial circulation, especially in the kidneys, causing retention of urine and the accumulation of the elements of the urine in the blood and tissues: to which may be added that Anaemia cerebri may also be caused by the same reflex imitation that causes contraction of the renal arterioles. Pathological Anatomy. Numerous as the cases are, and have been, of Parturient Eclampsia in women and cows, still, the results of microscopical examinations have been so unsatisfactory that we need not touch upon them in a paper devoted chiefly to its causes, as we find very little convincing with reference to the visible phenomona when nephritis is not present.

Prognosis. This is a most fatal complication in both Women and Cows: out of every three or four cases in the former one dies, and 40 to 50 per cent. of the latter.

Therapeutics. It is singular that whichever etiological theory we adopt, that the method of treatment is the same in both women and cows.

