# Correction

**Published:** 1884-04

**Authors:** 


Correction.—We beg to call attention to a serious typographical error on page 129, third line: the word "rare" having been mistaken for "sure."

